# Reference ranges for serum immunoglobulin (IgG, IgA, and IgM) and IgG subclass levels in healthy children

**DOI:** 10.3906/sag-1807-282

**Published:** 2019-04-18

**Authors:** Rumeysa Olcay BAYRAM, Hülya ÖZDEMIR, Ayça EMSEN, Hatice TÜRK DAĞI, Hasibe ARTAÇ

**Affiliations:** 1 Department of Pediatric Immunology and Allergy, Faculty of Medicine, Selçuk University, Konya Turkey; 2 Department of Medical Microbiology, Faculty of Medicine, Selçuk University, Konya Turkey

**Keywords:** Healthy children, IgG subclasses, reference interval, serum immunoglobulins

## Abstract

**Background/aim:**

The serum immunoglobulin levels are used routinely in clinical practice because they provide key information on the humoral immune status. This study aimed to determine the age-related reference values of serum immunoglobulin levels in healthy children.

**Materials and methods:**

A total of 330 healthy children, aged between 0 and 18 years, were included in this study. The serum immunoglobulin levels were measured using a nephelometric method in a total of 11 groups, each group consisting of 30 individuals, and IgG subclasses in 6 groups of children aged more than 2 years.

**Results:**

The serum IgG levels were high during the newborn period, decreased until the sixth month, and again increased to a maximum level at the age of 18 years. The level of IgA was found to be extremely low in the newborn period and then increased with age. While the lowest value was in the newborn period for serum IgM level, the highest value was in the 16- to 18-year-old period. The IgG subclasses varied depending on the age groups.

**Conclusion:**

The updated reference intervals of immunoglobulin levels in children may be used for the accurate diagnosis of immune deficiencies.

## 1. Introduction

The serum immunoglobulin levels are the most important screening tests for determining primary and secondary immunodeficiencies (1). They are also important in evaluating different clinical conditions such as autoimmune diseases and chronic liver diseases (2,3). Reference ranges according to age for immunoglobulins (IgG, IgA, and IgM) and IgG subclass concentrations (IgGSc) vary depending on the region, source population, applied method, and time. For this reason, these values need to be updated for an accurate diagnosis in the same ethnic group and same region. 

Primary immune deficiencies form a group of more than 350 diseases that affect the development and/or function of the immune system and are characterized by susceptibility to infections, autoimmune diseases and malignancy, and inherited gene defects. They are classified into nine different groups according to the component of the immune system that they affect (4). In pediatric clinical practice, severe, persistent, opportunistic or recurrent infections are the most frequent indication for determination of serum immunoglobulin levels. The knowledge of immunoglobulin isotype provides useful insight into complex humoral immune response. The antibody deficiencies are the most common types of the primary immunodeficiencies.****According to the European Society for Immunodeficiencies, the proportion of antibody deficiencies accounts for about 60% of all primary immunodeficiencies (5). This rate was found to be 73% in Turkey and 92% in the province of Konya in previous studies (6,7). 

The initial laboratory examination of humoral immunity consists of measuring levels of various immunoglobulin isotypes (IgG, IgA, IgM, and possibly IgGSc) in serum and comparison with values from normal age-matched controls. The assessment is clinically important when they are within the normal age-specific reference range. Because of the limited number of studies (8–10) and the necessity of an update, this study aimed to determine reference intervals in healthy children by measuring the levels of serum immunoglobulin and IgGSc.

## 2. Materials and methods

### 2.1. Study population

A total of 330 children without any diseases, between the ages of 0 and 18 years, were included in the study. During the first step for the study, serum samples of 540 healthy children were collected after written informed consent was obtained from all their parents. A form, which included information on sex, height, weight, date of birth, age at the time of admission, address and telephone information, the history of immune deficiency, allergic and chronic diseases was filled out. Of these serum samples collected over a 2-year period, 210 of the participants were removed from the study due to recurrent infections and various reasons including allergic, rheumatological, and gastrointestinal diseases detected during the children’s follow-up. Healthy children were divided into 11 groups according to age as follows: 30 newborns between 0 and 1 month; 30 children each between 1 and 3 months, 4 and 6 months, 7 and 12 months, 13 and 24 months, 25 and 36 months, 3 and 5 years, 6 and 8 years, 9 and 11 years, and 12 and 16 years, and 16 and 18 years. The serum immunoglobulin levels were measured using a nephelometric method in a total of 11 groups, each group consisting of 30 individuals, and IgG subclasses in 6 groups of children aged more than 2 years. This study was approved by the Ethical Committee of Selçuk University Medical Faculty.

### 2.2. Laboratory analysis

The levels of serum IgA, IgG, IgM, and IgG1, IgG2, IgG3, IgG4 were measured by the nephelometric method using the IMMAGE 800 device (Beckman Coulter, Inc., Brea, CA, USA). The values of serum immunoglobulin and IgG subgroup levels were expressed in mg/dL. The IMMAGE Immunochemistry Systems kits for IgA, IgG, and IgM and the kits for the IMMAGE 800 device (The Binding Site Group Ltd., UK) for the IgGSc were used. For measuring IgA, IgG, and IgM parameters (IMMAGE Immunochemistry Systems, Chemistry References, Beckman Coulter, Inc.) and IgGSc (The Binding Site Ltd.), an internal quality control study was performed using two levels of control serum. An international interlaboratory comparison program (RIQAS International Quality Assessment Scheme, UK) was followed for the external quality control.

### 2.3. Statistical analysis

SPSS for Windows 15.0 was used for statistical analyses. While the IgG, IgA, and IgM values were examined for the groups after 1–30 days, 1–3 months, 4–6 months, 7–12 months, 13–24 months, 25–36 months, 3–5 years, 6–8 years, 9–11 years, 12–16 years, and 16–18 years; the IgG1, IgG2, IgG3, and IgG4 values were measured for the groups 25–36 months, 3–5 years, 6–8 years, 9–11 years, 12–16 years, and 16–18 years. Descriptive statistics, such as geometric mean, arithmetic mean, minimum, and maximum values, and mean ± 2 standard deviation values for each age group, were given. A 95% confidence interval was also established for each age group. 

The data were divided into subgroups according to sex and age group. The difference between the age groups and sex (IgG, IgA, IgM, IgG1, IgG2, IgG3, and IgG4) was investigated. When comparing the differences between the two groups, the t-test was used in independent groups showing normal distribution and the Mann–Whitney U test was used for abnormal distribution. When comparing the mean between more than two groups, the one-way analysis of variance (ANOVA) was used for normal distribution and the Kruskal–Wallis test was used for the abnormal distribution. The significance level was accepted as P < 0.05.

## 3. Results

In this study, 177 of 330 patients were male and 153 were female. When serum immunoglobulins and IgGSc were evaluated according to sex, the serum IgM and IgG2 levels were higher in females (P = 0.004 and 0.011, respectively). The serum IgM levels were significantly different at the age groups including 1–30 days, 6–8 years, and 12–16 years (P = 0.04, P = 0.002 and P = 0.01, respectively). The serum IgG2 levels were higher in females at the age of 6–8 years and 16–18 years (P = 0.04 and P = 0.003, respectively). No significant difference in IgG, IgA, IgG1, IgG3, and IgG4 was observed between boys and girls (Table 1). 

**Table 1:** Examination of variables according to sex.

Variable	Test	P-value	Mean ± SD
IgG	t-test	0.673	No difference
IgA	Mann–Whitney U test	0.567	No difference
IgM	t-test	0.004	Difference present; higher in girls (1–30 days, 6–8 years and 12–16 years)*
IgG1	t-test	0.471	No difference
IgG2	Mann–Whitney U test	0.011	Difference present; higher in girls (6–8 years and 16–18 years)*
IgG3	Mann–Whitney U test	0.066	No difference
IgG4	Mann–Whitney U test	0.186	No difference

IgG: Immunoglobulin G, IgA: Immunoglobulin A, IgM: Immunoglobulin M * The difference in the serum IgM and IGG2 levels according to sex was detected in these age groups.

The difference in the IgG, IgA, IgM, and IgGSc according to age was statistically analyzed using the ANOVA and Kruskal–Wallis tests (Table 2). The geometric mean, arithmetic mean, standard deviation, minimum and maximum values, and 95% confidence intervals for the serum IgG, IgA, IgM, and IgGSc concentrations are shown in Tables 3–9. 

**Table 2:** Examination of variables according to age.

Variable	Test	P-value	Decision
IgG	ANOVA	0.000	Difference present (it increased with age, except for the 0- to 1-month age group; the highest value was found in the 16- to 18-year age range)
IgA	Kruskal–Wallis test	0.567	No difference
IgM	ANOVA	0.000	Difference present (while the highest value was found in the 16- to 18-year age range, the lowest was found in the 0- to 1-month age group)
IgG1	ANOVA	0.000	Difference present (while the highest value was found in the 16- to 18-year age range, the lowest was found in the 3- to 5-year age group)
IgG2	Kruskal–Wallis test	0.011	Difference present (the value increased with age)
IgG3	Kruskal–Wallis test	0.066	No difference
IgG4	Kruskal–Wallis test	0.186	No difference

ANOVA: Analysis of variance IgG; Immunoglobulin G, IgA: Immunoglobulin A, IgM: Immunoglobulin M

**Table 3:** Age-related serum IgG levels in healthy children (mg/dL).

Age group	Number	Geometric mean ± SD	Mean ± SD	Min–max	95% confidence interval
0–30 days	30	913.85 ± 262.19	953 ± 262.19	399–1480	855.1–1050.9
1–3 months	30	409.86 ± 145.59	429.5 ± 145.59	217–981	375.14–483.86
4–6 months	30	440.17 ± 236.8	482.43 ± 236.8	270–1110	394.01–570.86
7–12 months	30	536.79 ± 186.62	568.97 ± 186.62	242–977	499.28–638.65
13–24 months	30	726.79 ± 238.61	761.7 ± 238.61	389–1260	672.6–850.8
25–36 months	30	786.41 ± 249.14	811.5 ± 249.14	486–1970	718.47–904.53
3–5 years	30	823.19 ± 164.19	839.87 ± 164.19	457–1120	778.56–901.18
6–8 years	30	982.86 ± 255.53	1014.93 ± 255.53	483–1580	919.52–1110.35
9–11 years	30	1016.12 ± 322.27	1055.43 ± 322.27	642–2290	935.09–1175.77
12–16 years	30	1123.56 ± 203.83	1142.07 ± 203.83	636–1610	1065.96–1218.18
16–18 years	30	1277.20 ± 361.89	1322.77 ± 361.89	688–2430	1187.63–1457.9
Total	330	773.94 ± 361.76	852.92 ± 361.76	217–2430	813.75–892.1

IgG: Immunoglobulin G

### 3.1. Serum immunoglobulin levels (IgG, IgA, IgM)

**Figure 1:**
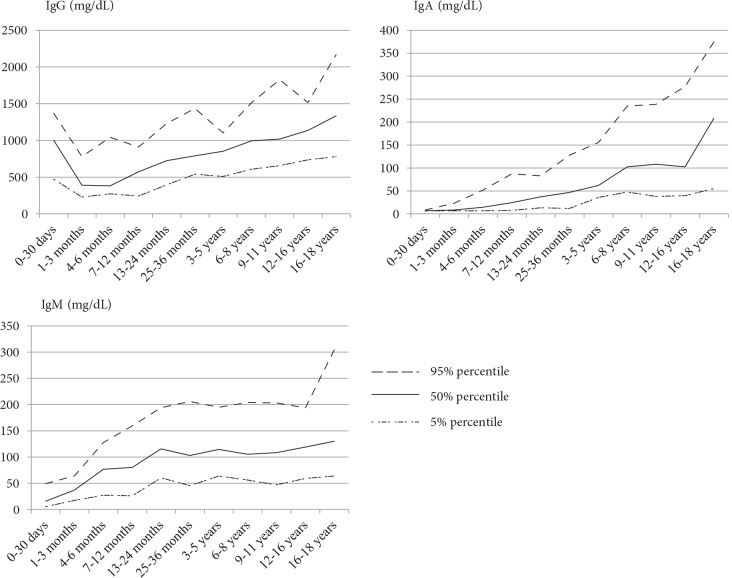
Percentile ranges of the serum IgG, IgA and IgM levels.

Age-related changes in the serum IgG, IgA, and IgM (the 5%, 50%, 95% percentile ranges) are shown in Figure 1. According to age groups, the serum IgG levels were found to be high in the newborn period (0–30 days) due to maternal IgG. These parameters were the lowest level due to loss of antibodies from the mother and inadequate synthesis in the second and third groups (1–6 months). Starting from the 6th month, the serum IgG levels increased, reaching maximum values at 16–18 years of age (Table 3). 

No significant difference was detected according to age for serum IgA levels. Although the IgA levels were very low during the neonatal period, they increased with age and reached their maximum value at the age of 18 years (Table 4).

**Table 4:** Age-related serum IgA levels in healthy children (mg/dL).

Age group	Number	Geometric mean ± SD	Mean ± SD	Min–max	95% confidence interval
0–30 days	30	6.77 ± 0.45	6.79 ± 0.45	6.67–8.75	6.62–6.95
1–3 months	30	9.58 ± 5.16	10.53 ± 5.16	6.67–24.6	8.57–12.49
4–6 months	30	17.23 ± 9.77	19.86 ± 9.77	6.67–53	14.70–25.01
7–12 months	30	23.63 ± 12.37	29.41 ± 12.37	6.68–114	21.06–37.77
13–24 months	30	34.09 ± 17.1	37.62 ± 17.1	13.1–103	31.34– 47.85
25–36 months	30	48.87 ± 24.52	59.77 ± 24.52	6.67–135	46.05–71.38
3–5 years	30	62.75 ± 34.05	68.98 ± 34.05	35.7–192	56.27–81.7
6–8 years	30	97.38 ± 49.66	106.9 ± 49.66	44.8–276	88.36–125.45
9–11 years	30	102.27 ± 47.05	115.99 ± 47.05	32.6–262	94.69–137.29
12–16 years	30	112.16 ± 47.51	120.90 ± 47.51	36.4–305	99.29–172.11
16–18 years	30	179.21 ± 89.92	201.84 ± 89.92	46.3–385	168.26– 235.41
Total	330	41.04 ± 32.13	70.78 ± 32.13	6.67–385	64.07–80.54

IgA: Immunoglobulin A

The IgM levels increased steadily after neonatal period but were low slightly in the 25- to 36-month group and after 6 years of age until the age of 11 years compared to the other age groups. After the age of 11 years, they increased again and reached their maximum value in the 16 to 18 year age group (Table 5).

**Table 5:** Age-related serum IgM levels in healthy children (mg/dL).

Age group	Number	Geometric mean ± SD	Mean ± SD	Min–max	95% confidence interval
0–30 days	30	16.89 ± 8.87	20.38 ± 8.87	5.1–50.9	15.57–5.18
1–3 months	30	34.21 ± 13.55	36.66 ± 13.55	15.2–68.5	31.60–41.72
4–6 months	30	69.05 ± 29.73	75.44 ± 29.73	26.9–130	64.34–86.54
7–12 months	30	73.42 ± 35.76	81.05 ± 35.76	24.2–162	67.7–94.41
13–24 months	30	115.25 ± 41.63	122.57 ± 41.63	38.6–195	107.03–138.12
25–36 months	30	104.66 ± 40.55	111.31 ± 40.55	42.7–236	96.17–126.46
3–5 years	30	115.60 ± 39.24	121.79 ± 39.24	58.7–198	107.13–136.44
6–8 years	30	108.05 ± 41.27	114.73 ± 41.27	50.3–242	99.32–130.14
9–11 years	30	104.95 ± 43.68	113.18 ± 43.68	37.4–213	96.87–129.49
12–16 years	30	119.16 ± 39.31	125.78 ± 39.31	42.4–197	111.1–140.46
16–18 years	30	130.60 ± 64.32	142.54 ± 64.32	60.7–323	118.53–166.55
Total	330	78.74 ± 43.46	96.86 ± 43.46	5.1–323	91.07–102.65

IgM: Immunoglobulin M

### 3.2. Serum immunoglobulin G subclass levels (IgG1, IgG2, IgG3, IgG4)

**Figure 2:**
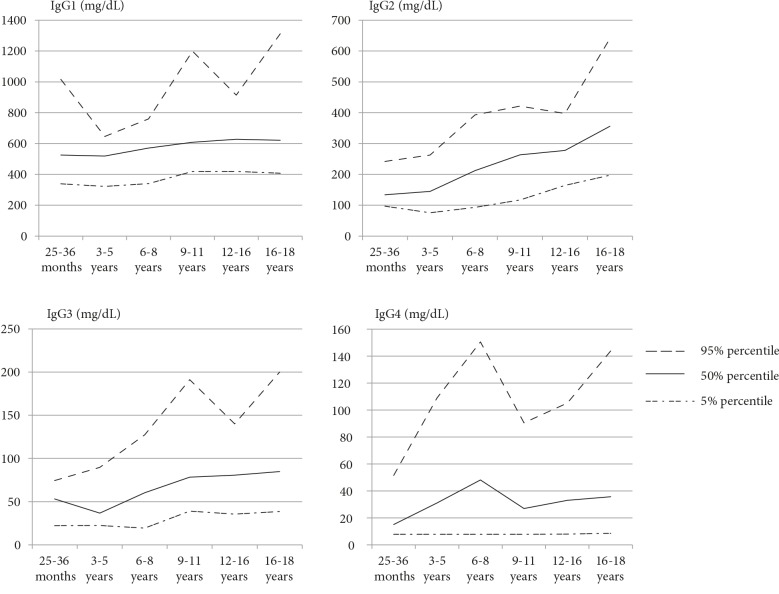
Percentile ranges of the serum IgG subclasses (IgG1, IgG2, IgG3 and IgG4) levels.

The 5%, %50, and %95 percentile ranges (in increasing order to levels in the graphs) for the IgG subclasses are shown in Figure 2. According to age, the highest value was in the 16 to 18 years of age range for serum IgG1 level, while the lowest value was in the 3 to 4 years of age. The serum IgG2 values increased with age. No statistically significant difference was observed in the serum IgG3 and IgG4 levels according to age (Tables 6–9). 

**Table 6:** Age-related serum IgG1 levels in healthy children (mg/dL).

Age group	Number	Geometric mean ± SD	Mean ± SD	Min–max	95% confidence interval
25–36 months	30	510.74 ± 192.04	531.7 ± 192.04	309–1450	459.98–603.41
3–5 years	30	506.73 ± 82.28	513.93 ± 82.28	273–679	483.20–544.65
6–8 years	30	567.94 ± 121.64	581 ± 121.64	292–781	535.87– 626.72
9–11 years	30	634.17 ± 216.39	660.23 ± 216.39	410–153	579.43–741.03
12–16 years	30	635.52 ± 131.01	648.53 ± 131.01	344–958	599.61–697.45
16–18 years	30	645.35 ± 229.62	674.5 ± 229.62	403–1520	588.75–760.24
Total	180	580.41 ± 179.97	601.7 ± 179.97	273–1530	575.22–628.17

IgG1: Immunoglobulin G1

The IgG1 levels were the lowest in the 25- to 36-month group and increased after 36 months until the age of 9 years. A second decline was seen in the 9- to 11-year age group, and another increase was seen at the age of 12–16 years. After the age of 16 years, they reached the adult level with a third decline (Table 6). 

The IgG2 levels increased from the 25th month onward and reached maximum values at the age of 16–18 years (Table 7).

**Table 7:** Age-related serum IgG2 levels in healthy children (mg/dL).

Age group	Number	Geometric mean ± SD	Mean ± SD	Min–max	95% confidence interval
25–36 months	30	137.88 ± 38.59	141.98 ± 38.59	87.6–289	127.57–156.39
3–5 years	30	143.92 ± 50,802	151.95 ± 50.80	73.3–271	132.98–170.92
6–8 years	30	196.57 ± 86.41	213.67 ± 86.41	88.1–408	181.40– 245.93
9–11 years	30	250.67 ± 85.72	265.56 ± 85.72	81–442	233.55–297.57
12–16 years	30	261.62 ± 69,147	270.23 ± 69.14	159–406	244.41–296.05
16–18 years	30	359.76 ± 115,839	375.9 ± 115.83	184–696	332.6–419.15
Total	180	212.48 ± 111,265	236.55 ± 111.26	73.3–696	220.18–252.91

IgG2: Immunoglobulin G2

The IgG3 levels showed a partial decline at the age of 6–8 years and then continued to increase steadily. The levels reached a maximum value at the age of 16–18 years (Table 8).

**Table 8:** Age-related serum IgG3 levels in healthy children (mg/dL).

Age group	Number	Geometric mean ± SD	Mean ± SD	Min–max	95% confidence interval
25–36 months	30	15.53 ± 8.54	18.37 ± 8.54	7.86–57.5	13.67–23.07
3–5 years	30	30.81 ± 15.42	40.75 ± 15.42	7.86–122	28.84–52.65
6–8 years	30	39.33 ± 23.05	50.94 ± 23.05	7.86–157	37.11–64.77
9–11 years	30	25.36 ± 15.39	35.51 ± 15.39	7.86–93.8	24.95–46.07
12–16 years	30	31.03 ± 16.73	39.51 ± 16.73	7.86–119	29.53–49.49
16–18 years	30	38.89 ± 23.08	50.16 ± 23.08	7.86–157	36.32–64.01
Total	180	28.71 ± 15.76	39.21 ± 157.66	7.86–157	34.56–43.86

IgG3: Immunoglobulin G3

Although the IgG4 levels showed fluctuations at the age of 2–11 years, they continued to increase after the age of 11 years and reached the maximum values in the 16- to 18-year age group (Table 9).

**Table 9:** Age-related serum IgG4 levels in healthy children (mg/dL).

Age group	Number	Geometric mean ± SD	Mean ± SD	Min–max	95% confidence interval
25–36 months	30	48.78 ± 16.90	51.73 ± 16.90	19.8–75	45.41–58.04
3–5 years	30	44.05 ± 21.55	45.26 ± 21.55	20.8–93.2	37.21–53.30
6–8 years	30	56.82 ± 30.55	65.53 ± 30.55	18.9–135	53.01–78.06
9–11 years	30	77.59 ± 37.37	84.19 ± 37.37	34.1–200	70.24–98.15
12–16 years	30	75.30 ± 31.86	81.39 ± 31.86	35.2–150	69.49–93.28
16–18 years	30	86.33 ± 43.29	95.12 ± 43.29	29.3–200	78.95–111.28
Total	180	62.72 ± 37.13	70.53 ± 30.13	18.9–200	65.19–75.837

IgG4: Immunoglobulin G4

## 4. Discussion

The first stage in evaluating possible underlying immunodeficiency is the measurement of blood count and serum immunoglobulin levels (11). The standards for changes in antibody production by age during childhood vary according to different ethnic groups and settlement regions. Hence, the immunoglobulin levels should be compared with age-appropriate reference values according to the 95% confidence interval. In this study, the distribution of serum immunoglobulins and IgGSc according to age and sex were examined in children and the reference intervals were determined. 

In the present study, the serum IgG levels were extremely high in the newborn period due to the IgG passed on from the mother. These parameters fell to the lowest level due to loss of antibodies from the mother and inadequate synthesis in the baby in the second and third groups (1–6 months). Again, the serum IgG levels increased with age, starting from the 6th month, reaching maximum values at 16–18 years of age. However, the IgA levels were extremely low in the newborn period and increased with age, reaching their maximum values at the age of 18 years. These results were compatible with the findings of studies conducted in Turkey and quoted in the literature (8–10, 12). The IgM levels did not show a continuous increase in our study, contrary to the previous studies (8–10, 12). The continuous increase seen in the 1st month fluctuated till 2 years of age. Low values were determined in the 25- to 36-month group, and then from the ages of 6 years until 11 years. After the age of 11 years, they increased again and reached their maximum value in the age group of 16 to 18 years.

Previous studies explored the normal values of immunoglobulin levels which obtained by different methods, such as enzyme-linked immunosorbent assay (ELISA), radioimmunoassay, turbidimetry, and nephelometry, in children and adults (12–14). The International Federation of Clinical Chemistry and Laboratory suggests that each laboratory should calculate its own reference values and publish, together with the National Committee for Clinical Laboratory Standard, guiding standards for laboratories (15). Studies on reference intervals of serum immunoglobulins and IgGSc, which were used in the diagnosis and follow-up of various immunological diseases, demonstrated that these values changed depending on the methods used, patient population, age, and sex. The inconsistencies seen in some of the earlier studies might be due to the method of analysis. In the earlier studies, the serum immunoglobulins and IgGSc were measured using radial immunodiffusion, which was then changed with a method developed based on ELISA using monoclonal antibodies. Nowadays, the nephelometric measurement method, which is faster and has less variability, is more commonly used (14,16). In the present study, the immunoglobulin levels were measured by the nephelometric method. 

The first study performed on this subject in Turkey was in 1996 in Ankara, where the normal values of the IgG, IgA, and IgM levels were determined using the turbidimetric method. The serum IgG fell to 39% of the adult level after 31 days. It was 52% at the age of 1 year, increased to 97% of the adult level at the age of 7 years, and elevated above the adult level between the ages of 11 and 16 years (8). Tezcan et al. observed a continuous increase in the IgM levels in the 1st month, reaching close to the adult level at early ages. Between the ages of 13 and 16 years, the levels reached the adult levels. Although the IgA levels were extremely low during the neonatal period, they increased with age and passed 90% of the adult level at the age of 13 years. These results were consistent with the findings of our study.

The second study was conducted in 2006 by Aksu et al. using the nephelometric method, and the reference ranges of IgG, IgA, IgM, and IgGSc were determined. In that study, the levels of serum IgG and IgGSc were higher in the newborn period (0–30 days) due to maternal IgG. In children in the second group (1–5 months), these parameters fell to the lowest level due to loss of antibodies from the mother and inadequate synthesis in the baby. After the 6th to 8th month, an increase in immunoglobulins and IgGSc according to age was observed. The IgG, IgA, IgG1, and IgG2 levels increased until the age of 9–10 years, and then reached a plateau. The IgG3 and IgG4 levels increased up to the ages of 7–8 years and then reached a plateau. The IgM levels increased earlier and reached a balanced value after the first year of life (9). In our study, all immunoglobulin levels increased with age and no plateau was observed.

The third study was carried out in İzmir in 2010 using the nephelometric method, and the reference ranges of IgG, IgA, IgM, and IgGSc were determined. In females, the reference intervals of all tests were found to be higher in females compared with males (10). No newborn separation was done, and neonates were evaluated after 0–6 months. In that****study, the IgG values in the 95% confidence interval were found to be lower in the first 12 months and then increased in the later age groups. This might be due to the fact that fewer data were available on the age groups in that study and statistical analysis was lacking.

The IgG subclass deficiencies are defined as one or both of the IgGSc below normal values according to age, while the IgG, IgA, and IgM values are normal. Studies have shown that IgG1 and IgG3 levels reach 75% of the adult level at 5 years of age. The level of IgG2 reaches 75% of the adult level at 14 years of age. However, the normal limits of IgG4 could not be fully determined and IgG4 deficiency should not be diagnosed under the age of 10 years (1). In our study, the level of IgG1 reached 76% of the 16- to 18-year age group values at the age of 3–5 years, and the level of IgG2 reached approximately 70% of the 16- to 18-year age group level between 9 and 11 years of age and the level of IgG3 between the ages of 6 and 8 years. The level of IgG4 the 3- to 5-year age group reached 80% of the biggest childhood age group. IgG4 is a blocking antibody and does not bind to its complement. Its level decreases in allergic diseases and increases with allergen immunotherapy. We observed that the adult levels can be reached earlier in the population used in this study. It is also important for determining the upper limits that regulate IgG4-related diseases. 

Several studies have reported IgGSc in healthy children and adults (17,18). We observed the IgG1, IgG2, and IgG3 levels increased with age. The maximum reference values for all IgGSc were reached in the 16- to 18-year age group. The results were broadly similar to those reported in previous studies. Fluctuations in IgG4 levels were observed between the age groups in our study, whereas IgG4 concentrations continued to increase with age in some studies (18). Our results are similar to the findings of a Thai study (19). 

The present study had some limitations. Our population included healthy children who live in Konya and therefore the selected samples may not totally reflect the Turkish population. However, Konya is a big city in the Central Anatolia including admixture of Turkish children, so we think that the updated reference values may be used in Turkey. The relatively small study population may reduce the power of the study and the national database is needed for serum immunoglobulin levels in a larger Turkish population.

In conclusion, the present study provided usable data on normal values and age-related changes in serum immunoglobulins and IgGSc. It constituted a step in establishing a reliable reference source for laboratories. It made an important contribution, especially in determining the reference values of regional immunoglobulins and IgGSc, for the childhood age group. Previous studies suggested that issues, such as difficulties encountered in identifying reference individuals, limitations of small laboratories, and standardization of regional and biological variations, need to be resolved with multicentered and national reference policies. A healthy regional database should be maintained with the participation of larger sample groups and by continuing these studies. The reference values for serum immunoglobulin levels and IgGSc in healthy children were updated with this study. Autosomal recessive inherited primary immune deficiencies are common in Turkey with high rates of consanguineous marriages. This study is important in terms of being a reference source for assessing immunoglobulin levels for accurate diagnosis, treatment, and follow-up of primary and secondary immunodeficiencies in children.

## Acknowledgments

We thank the children and their families for participating. The study was supported by the Scientific Research Projects of Selçuk University (Project No: 15102034).
